# SIX MONTHS OF FOCUSED EXERCISE IN GEROFIT LEADS TO REDUCED FALL RISK THROUGH IMPROVED BALANCE

**DOI:** 10.1093/geroni/igz038.137

**Published:** 2019-11-08

**Authors:** Arti S Tayade, Jody R Colglazier, Alan D Wesley, Paul J Froehlich, Elizabeth C Hersch, Anne P Poppe, David E Hall, Suzanne M Younker

**Affiliations:** 1 VA Puget Sound, Seattle, Washington, United States; 2 VA Puget Sound Health Care System, Lakewood, Washington, United States; 4 Greenriver Community College, Tacoma, Washington, United States; 3 VA Puget Sound Health Care System, Seattle, Washington, United States

## Abstract

Many older Veterans are physically debilitated, with a reduced quality of life (QOL.) Use of assistive devices during ambulation is indicative of fall risk. We describe two case studies following six-months of participation in Gerofit, a structured exercise program. Ms. M., a 75 y/o Veteran with chronic PTSD and joint pain could walk 237 yards in six-minutes using a cane. Mr. D., an 86 y/o Veteran, came to Gerofit using a Front Wheeled Walker and could walk 307 yards in 6-minutes. After six-months of Gerofit, neither Veteran used assistive devices during testing, and both reported higher QOL. Ms. M. now walks 514 yards in six minutes, while Mr. D. can walk 375 yards in six minutes. Both Veterans exhibit improved balance and endurance. They tell us: “Gerofit gave me my life back!” and “Gerofit gets me out of the house and off the couch.”

